# The Impact of Psychobiotic Therapy on Anxiety and Depressive Symptoms in Patients with Post-COVID Syndrome—A Randomized, Double-Blind, Placebo-Controlled Trial

**DOI:** 10.3390/metabo16080594

**Published:** 2026-08-19

**Authors:** Sylwia Drzymała, Anna Blask-Osipa, Anna Szczepańska-Álvarez, Małgorzata Dobrzyńska, Hanna Markowska, Sławomira Drzymała-Czyż, Jarosław Walkowiak

**Affiliations:** 1 Department of Pediatric Gastroenterology and Metabolic Diseases, Poznan University of Medical Sciences, Szpitalna 27/33, 60-572 Poznan, Poland; 2 Complex of Healthcare Institutions, Kościuszki 96, 64-700 Czarnków, Poland; 3 Department of Mathematical and Statistical Methods, Poznan University of Life Sciences, Wojska Polskiego 28, 60-637 Poznan, Poland; 4 Department of Bromatology, Poznan University of Medical Sciences, Rokietnicka 3 Street, 60-806 Poznan, Poland

**Keywords:** probiotics, *Lactobacillus helveticus*, *Bifidobacterium longum*, SARS-CoV-2

## Abstract

**Background**: Recovering from COVID-19 does not always lead to complete recovery, and many patients report symptoms of anxiety and depression. One potential mechanism behind these disorders is dysbiosis. The aim of this study was to evaluate the impact of psychobiotic therapy on the effectiveness of treating anxiety and depressive symptoms in individuals who had previously experienced COVID-19. **Methods**: This study was designed as a randomized, double-blind, placebo-controlled trial. A total of 62 participants were randomly allocated to one of two groups: a psychobiotic group (PG; 23 women, 9 men) receiving 3 × 10^9^ CFU *Lactobacillus helveticus* and *Bifidobacterium longum*, or a control group (CG; 21 women, 9 men) receiving a placebo, for 6 weeks. Fifty-six participants finalized the study and were included in the analysis (28 per group). The severity of depressive and anxiety symptoms at baseline and after the intervention was assessed using validated self-assessment tools: the Hospital Anxiety and Depression Scale (HADS), the Generalized Anxiety Disorder 7-item scale (GAD-7), the Beck Depression Inventory (BDI), and the Hopkins Symptom Checklist (SCL-90). Additionally, the profile of selected short-chain fatty acids (SCFAs) in the stool was assessed. **Results**: Following the supplementation period, the PG showed significantly lower median levels of depression and anxiety compared to the CG, corresponding to a 57.2% reduction in anxiety severity (GAD-7) and a 50.9% reduction in depressive symptoms (BDI) (HADS—Depression and Anxiety, and GAD-7: *p* = 0.0001; BDI: *p* = 0.0003). Additionally, the prevalence of anxiety and depressive symptoms significantly decreased in the PG compared to the CG based on the HADS-A (*p* = 0.0044), GAD-7 (*p* = 0.0219), and BDI (*p* = 0.0011) scores. Among the measured SCFAs, only the butyric acid concentration in PG increased significantly during supplementation (baseline vs. follow-up—*p* = 0.0338) and was higher in PG than in CG at the study’s end (*p* = 0.0248). However, in the PG, changes in the concentrations of all analyzed acids correlated with a reduction in the severity of anxiety symptoms measured using the HADS-A. **Conclusions**: Psychobiotic supplementation in individuals recovering from COVID-19 who exhibit anxiety and/or depressive symptoms led to mood improvement and a reduction in symptom severity, and it may serve as a supportive treatment.

## 1. Introduction

The SARS-CoV-2 pandemic has contributed to a decrease in people’s mental fitness and resilience worldwide [1,2]. On one hand, social isolation caused by quarantine, the need to limit personal contact and adhere to strict epidemic rules, and fear for one’s own health and that of one’s loved ones already act as stressors [3,4]. On the other hand, the infection itself may cause neurological and mental complications [5]. Chronic neuropsychiatric consequences of SARS-CoV-2 infection include depression, anxiety, psychotic disorders, demyelinating complications [6]. Potential mechanisms that lead to the development of the above-mentioned symptoms include the infiltration of the virus into the central nervous system, dysregulation of the cytokine network, migration of peripheral immune cells, and translocation of intestinal microorganisms [7,8]. Studies conducted so far have shown that viral RNA excretion in the feces of COVID-19 patients persists for up to 7 months after infection [9]. Although the extent and mechanisms of viral infiltration of the intestinal epithelium are not yet known, almost 40% of patients with COVID-19 have gastrointestinal symptoms accompanied by changes in the composition of the intestinal microbiota [10,11]. It is worth emphasizing that dysbiosis may also contribute to the pathogenesis of neuropsychiatric symptoms via the gut–brain axis. Changes in microbiota composition can contribute to increased intestinal permeability, exacerbate gastrointestinal inflammation, and disrupt gut–brain axis function [12]. Dysbiosis also increases pro-inflammatory cytokine production and enhances glutamic acid release, which, as a neurotransmitter, reduces brain-derived neurotrophic factor (BDNF) production, resulting in decreased neuronal plasticity and lower serotonin production [13]. Therefore, gut dysbiosis appears to contribute to the development of anxiety and depressive symptoms in patients who have gone through COVID-19, especially those with post-COVID syndrome [14]. This underscores the rationale for supplementing these patients with specific probiotics.

Psychobiotics are defined as probiotic preparations that, when consumed in appropriate amounts, interact with gut bacteria to benefit the body and support mental health by influencing the hypothalamic–pituitary–adrenal (HPA) axis [15,16]. However, the beneficial effects of psychobiotics extend beyond mental health to the gastrointestinal system. Firstly, like other probiotics, psychobiotics can beneficially modify gut microbiota by competing with pathogenic microorganisms for adhesion sites on the intestinal epithelium and for essential nutrients [17,18]. Secondly, they exert a direct influence on the gut-associated lymphoid tissue (GALT), regulating the pro-inflammatory response in Peyer’s patches, activating the synthesis of anti-inflammatory cytokines, inhibiting pro-inflammatory compound production as a result of changes in the T-lymphocyte population, and interacting with dendritic cells, macrophages, and B lymphocytes [19]. Thirdly, and most importantly, psychobiotics can produce a range of bioactive molecules, including neurotransmitters and neuromodulators such as γ-aminobutyric acid, 5-hydroxytryptamine, and noradrenaline [20,21]. Studies evaluating the potential of psychobiotics are becoming increasingly common, and literature reviews suggest that psychobiotic consumption may be considered a viable option to support the treatment of anxiety and depressive symptoms [22,23,24]. The above rationale enabled us to formulate the following research hypothesis: supplementation with the psychobiotics *Lactobacillus helveticus* and *Bifidobacterium longum* would reduce the severity of anxiety and depressive symptoms and favorably affect fecal SCFA concentrations in individuals experiencing anxiety–depressive symptoms associated with post-COVID syndrome. Therefore, the aim of the present study was to evaluate the effect of supplementation with these psychobiotics on the severity of anxiety and depressive symptoms in individuals with post-COVID syndrome, as well as its impact on fecal SCFA concentrations. The findings of this study may contribute to the development of supportive therapeutic strategies for this patient population.

## 2. Materials and Methods

### 2.1. Study Overview

This study was designed as a randomized, double-blind, placebo-controlled trial [25]. All patients provided written informed consent to participate in the study, and the study received approval from the Bioethics Committee of the Poznan University of Medical Sciences (No. 665/21). The study was registered in the German Clinical Trials Register (DRKS00026955; date of registration: 22 October 2021), which is part of the primary network of the World Health Organization. The study was conducted in accordance with CONSORT 2025 standards.

### 2.2. Participants

The study included adult subjects who had gone through COVID-19 and, based on psychological tests, were diagnosed with emerging post-COVID depressive and/or anxiety disorders. Patients were recruited among individuals admitted to the post-COVID department of the Complex of Healthcare Institutions in Czarnków, Poland, which served as an infectious disease hospital during 2021–2022. Patients who met the predefined inclusion and exclusion criteria were invited to participate in the study. Participants were enrolled approximately 5–6 months after acute SARS-CoV-2 infection. Specifically, 92% of participants were assessed in the fifth month after infection, while the remaining 8% were assessed in the sixth month. Post-COVID-19 syndrome symptoms were assessed in each participant, following the guidelines of the National Institute for Health and Care Excellence (NICE). Patients were always included in the study by the same physicians (ABO and HM) and psychologist (SD) throughout the study. The inclusion criteria were: age 18–65 years, self-reported post-COVID depressive or anxiety symptoms (confirmed by psychological testing—a score of ≤7 Sten on the Goldberg General Health Questionnaire (GHQ)), and consent to participate in the study.

The exclusion criteria included the following: pregnancy; severe comorbid diseases (liver cirrhosis, portal hypertension, renal failure, oncological diseases, type I diabetes, connective tissue diseases); previously (pre-COVID) diagnosed psychiatric disorders; psychiatric treatment; the use of psychotropic medications; intake of dietary supplements with stimulating/activating properties (ginger, guarana, ginseng, melatonin, etc.); and probiotics. Individuals whose mental state required immediate psychiatric assistance were also excluded from the study. All patients were informed about the safety of using psychobiotics. The patients were under the constant care of the psychologist (SD) and the physician (ABO).

### 2.3. Intervention

After randomization, patients were randomly assigned to one of two groups. The psychobiotic group (PG) received one capsule containing 3 × 10^9^ CFU of *Lactobacillus helveticus* Rosell^®^–52 and *Bifidobacterium longum* Rosell^®^–175, whereas the control group (CG) received an identical placebo capsule containing maize starch and maltodextrins. Psychobiotics and placebo capsules were supplied by SANPROBI Sp. z o.o. Sp. K. (Szczecin, Poland). The strains used in the study were granted a safety status—known as QPS (Qualified Presumption of Safety)—by the European Food Safety Authority (EFSA) [26]. The International Dairy Federation (IDF), in collaboration with the European Food and Feed Cultures Association (EFFCA), included them in the list of microorganisms with a documented history of safe use in foods [27].

The randomization was supervised by a study assistant who was not involved in the research. A randomization table was generated using the website randomizer.org (allocation ratio: 1:1). All study participants and investigators (psychologists, physicians, nurses, and the study coordinator) were blinded to the psychobiotics administered. All eligible participants were instructed to maintain their current diet throughout the experiment, abstain from taking probiotics (whether as supplements or medications), and avoid consuming foods enriched with probiotics or prebiotics. Participants were asked to take the supplement with breakfast (1 capsule each day). Supplementation lasted for 6 weeks. Adherence to the recommended regimen was monitored based on the number of blister packs returned at the final study visit.

### 2.4. Outcomes

The main outcome of the study was the change in the levels of anxiety/depression perceived by participants relative to baseline, measured using the Polish version of the Hopkins Symptom Checklist (primary endpoint) or other scales (Generalized Anxiety Disorder, Beck Depression Inventory, and Polish version of the Hopkins Symptom Checklist)—psychological tests are mentioned below (secondary endpoints).

### 2.5. Psychological Tests

For all individuals included in the study, the severity of depressive and anxiety symptoms was assessed using validated self-assessment scales:

- HADS (The Hospital Anxiety and Depression Scale) [28]: This questionnaire comprises two independent subscales for evaluating anxiety and depression, with each subscale containing seven items. Responses are provided on a 4-point Likert scale (0–3). The final score for each subscale ranges from 0 to 21 points. Scores between 0 and 7 indicate normal levels, scores from 8 to 10 suggest a borderline condition, and scores from 11 to 21 are considered abnormal.
- GAD-7 (Generalized Anxiety Disorder) [29,30]: This screening tool is used for the initial detection of anxiety disorders and for determining qualification for specialized treatment. The questionnaire is a brief self-report scale designed to identify probable anxiety disorders. It consists of seven questions pertaining to behavior and well-being over the past two weeks. Based on the obtained scores, the following interpretations are made: 0–4 indicates minimal anxiety; 5–9, mild anxiety; 10–14, moderate anxiety; and 15–21, severe anxiety.
- BDI (Beck Depression Inventory) [31,32]: This self-report instrument is used to assess the severity of depressive symptoms. The test consists of 21 questions concerning various aspects of depression, such as sadness, loss of interest, feelings of guilt, difficulties in concentration, and sleep disturbances. Respondents rate the degree to which each symptom applies to them on a 4-point scale: 0—not at all, 1—moderately, 2—markedly, and 3—very markedly. The sum of the scores indicates the overall severity of depression, interpreted as follows: 0–9 points signify no or minimal depression; 10–18 points indicate mild depression; 19–29 points suggest moderate depression; and 30–63 points denote severe depression.
- SCL-90 (Polish version of the HSCL 90, Hopkins Symptom Checklist) [33,34]: This standardized instrument assesses the severity of psychopathological symptoms. It comprises 90 items addressing nine key symptom clusters (somatization, obsessive–compulsive, interpersonal sensitivity, depression, anxiety, anger–hostility, phobic anxiety, paranoid ideation, and psychoticism). For each item, the patient selects one of five points that best reflects their feelings over the past 4 weeks. An average score of greater than 2 points for any group of items is considered high.

Psychological assessments were conducted according to the guidelines provided by the individual instrument developers. Assessments took place in a private room in the hospital and were conducted individually, with only the participant and a psychologist (SD) present. Before administration, participants were explained the study’s objectives and assured of the confidentiality of their responses. The psychologist addressed any questions arising during the assessment. Participants were not time-limited and could take short breaks between questionnaires if necessary; however, all assessments were completed on the same day. After completion, questionnaires were checked for missing responses to ensure data completeness.

### 2.6. Stool Short-Chain Fatty Acid (SCFA) Analysis

The determination and quantification of fatty acids traditionally classified as SCFA in feces was performed by gas chromatography with flame ionization detection (GC-FID), as previously described [35]. Fecal samples were collected twice, before and after the intervention. Thawed stool samples (100 mg) were homogenized and acidified with 50% sulfuric acid. After centrifugation, 50 μL of internal standard solution (IC6, 330 μM) was added. SCFA were extracted three times with 1 mL portions of diethyl ether, and then each extraction was centrifuged (5 min, 2800× *g*); the combined organic phases were collected. A 0.5 μL aliquot of the combined organic extract was injected into a gas chromatograph (Agilent 7890 Series II, Agilent Technologies, Santa Clara, CA, USA) equipped with a BPX-70 column (25 m × 0.22 mm × 0.25 μm; SGE Analytical Science, Ringwood, Australia). Compound identification was confirmed by mass spectrometry (Agilent 5975C, Agilent Technologies, Santa Clara, CA, USA), and peak integration was performed using an MSD ChemStation (Agilent Technologies, Santa Clara, CA, USA). Acid concentrations are given in µmol/g.

### 2.7. Minimum Sample Size

The sample size calculation was based on the expected change in the Anxiety subscale score of the SCL-90. A difference in the range of 1.5 to 2 points was considered significant (with a 3-point difference assumed). To detect this treatment effect with 80% power at a 5% significance level, it was necessary to include 28 participants in each group. Assuming a 10% dropout rate, a total of 62 subjects were required for the study. These calculations were performed using the G Power software (version 3.1.9.7) (University of Düsseldorf, Düsseldorf, Germany).

### 2.8. Statistical Analysis

All results were subjected to statistical analysis. Data are presented as medians with the 1st through 3rd quartiles and means (±standard deviations—SDs) with 95% confidence intervals (95% CI). After defining the lack of normality in the data distribution using the Shapiro–Wilk test, the Mann–Whitney U test was employed to compare results between groups. The correlation between changes in SCFAs concentrations and changes in the severity of psychopathological symptoms was analysed using Spearman’s test. Additionally, a chi-square test with Yates’ correction for continuity was conducted to examine the impact of the psychobiotic intervention on the psychological test outcomes. Hypotheses were tested at a significance level of 0.05. Statistical analysis was performed using GraphPad Prism 5.01 software (GraphPad Software, Inc., La Jolla, CA, USA) and Statistica 13.0 software (TIBCO, Palo Alto, CA, USA).

## 3. Results

**Figure 1:**
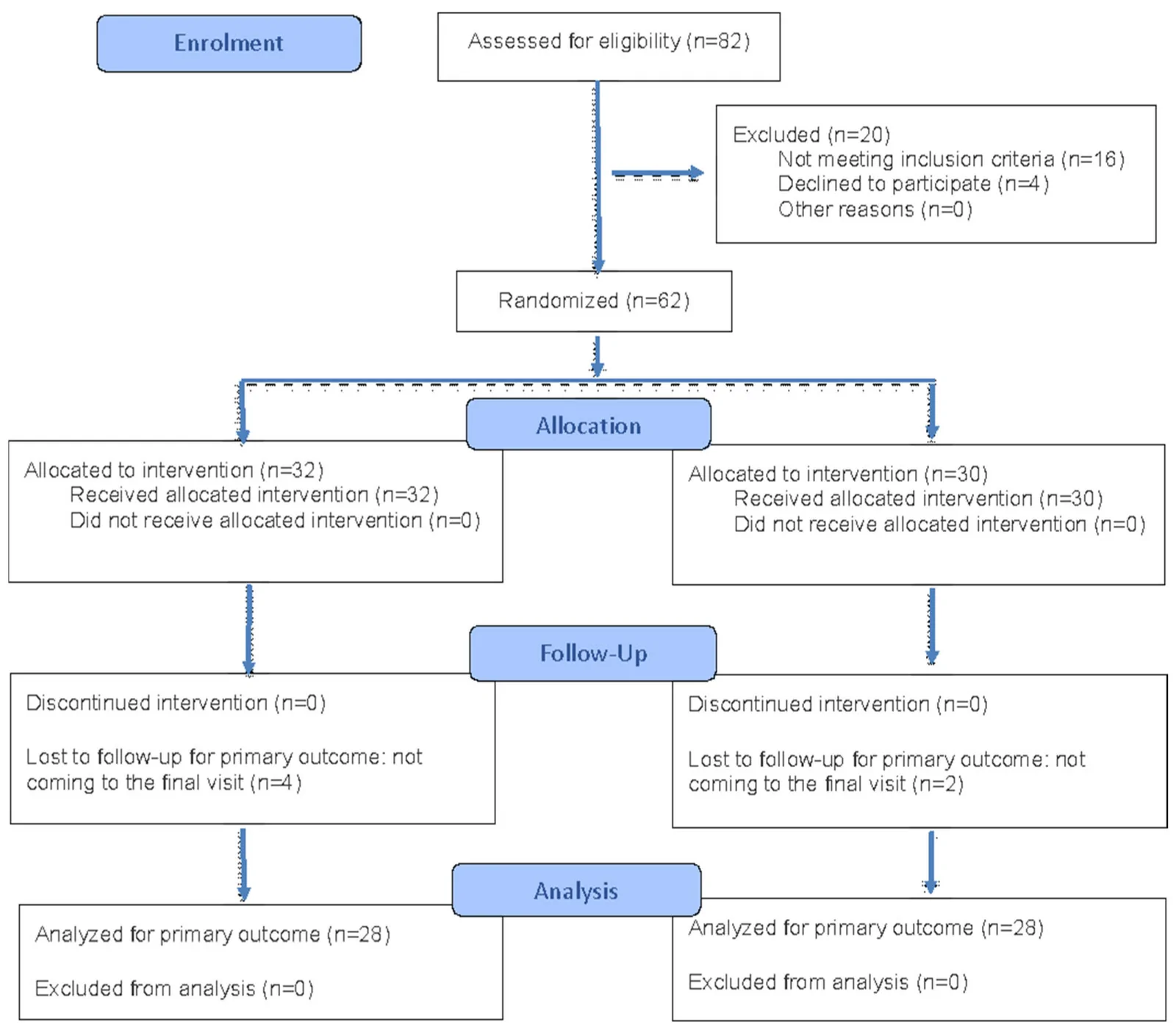
Participant flow diagram according to the CONSORT 2025 statement [36].

A total of 62 participants were randomized: 32 were allocated to the PG (23 women and 9 men) and 30 to the CG (21 women and 9 men). Ultimately, 56 patients completed the study (90.3%). Six individuals did not attend the visit after the supplementation period ended, without providing a reason for discontinuing the study (four women—one from the CG and three from the PG—and two men—one from the PG and one from the CG). None of the patients who completed the study reported any adverse events. Figure 1 presents the participants’ flow according CONSORT 2025.

The characteristics of the patients from both groups included in the study are presented in Table 1. The majority of the patients were individuals over 50 years of age who were overweight.

At baseline, the median levels of depression and anxiety measured by the HADS-D, HADS-A, GAD-7, and BDI scales did not differ significantly between the groups (Table 2). However, after the supplementation period, the median levels of depression and anxiety were significantly lower in the PG, and the difference in their severity measured at follow-up versus baseline (Δ follow-up—baseline psychobiotic group vs. Δ follow-up—baseline control group) was greater in the PG. Similarly, the Global Severity Index (GSI) of the SCL-90 did not differ significantly between groups at baseline. Following the intervention, the GSI was significantly lower in the PG than in the CG (*p* = 0.01), and the reduction from baseline (Δ follow-up—baseline) was significantly greater in the psychobiotic group (*p* < 0.0002).

The median severity level of each assessed psychopathological symptom, measured by the SCL-90 at baseline, did not differ significantly between the groups (Table 3). However, the median levels of interpersonal sensitivity, depression, anxiety, and paranoia measured after the supplementation period were statistically significantly lower in the PG, and the change in the severity of depression and anxiety from baseline to follow-up (Δ follow-up—baseline psychobiotic group vs. Δ follow-up—baseline control group) was greater in the PG.

Based on the established cut-off points in the applied tests, a diagnosis of anxiety or depression was made, and the number of individuals with this diagnosis was subsequently evaluated at baseline and after the supplementation period (Table 4). A statistically significant decrease in the prevalence of anxiety disorders assessed by the HADS-A test (*p* = 0.0044) and GAD-7 (*p* = 0.0219), as well as depressive disorders assessed by the BDI (*p* = 0.0011), was observed after the supplementation period in the PG. Due to the low prevalence of cases in the contingency table, χ^2^ analysis was not conducted for the other psychological tests, as the results might have been non-informative.

The median concentrations of all three SCFAs, namely acetic acid, propionic acid, and butyric acid, did not differ significantly between groups at baseline. Only butyric acid concentration in PG increased significantly during supplementation (baseline vs. follow-up—*p* = 0.0338) and was higher in PG than in CG at study end (follow-up vs. follow-up—*p* = 0.0248). In this group, a significant increase in butyric acid concentration during the supplementation period was also observed (ΔFU − BL vs. Δ FU − BL—*p* = 0.0064; Table 5).

In the PG, changes in the concentrations of each analyzed SCFA correlated with a reduction in the severity of anxiety symptoms measured using the HADS-A (Δ acetic acid vs. Δ HADS-A—*p* = 0.0007, rho = −0.6012, Δ propionic acid vs. Δ HADS-A—*p* = 0.0013, rho = −0.5771, Δ butyric acid vs. Δ HADS-A—*p* = 0.0008, rho = −0.5987, respectively). No associations between fatty acid concentration and psychopathological symptoms were observed in the CG.

## 4. Discussion

The results of our randomized, double-blind clinical trial—in which patients with anxiety–depressive symptoms received either a psychobiotic or a placebo for six weeks—unequivocally demonstrated that the administration of *Lactobacillus helveticus* Rosell^®^-52 and *Bifidobacterium longum* Rosell^®^-175 significantly improved patients’ mood and reduced symptom severity, as confirmed by psychological assessments using the HADS, GAD-7, BDI, and SCL-90 tests. It is important to emphasize that anxiety–depressive disorders, especially those occurring during the course of COVID-19 or the course of post-COVID-19 syndrome, are among the most frequently encountered mental disorders [37]. However, their appearance does not necessarily indicate a mental illness; if they are mild and do not limit daily functioning, there is no indication for the use of psychotropic medications. Psychobiotics can be helpful in such cases [38,39,40,41].

The results of our study are generally consistent with previous clinical research evaluating the effects of probiotic supplementation on mental health that focused on outcomes related to psychological well-being, including mood, stress, sleep quality, anxiety, and depressive symptoms [42,43,44,45,46,47,48,49,50,51,52]. Current research clearly indicates that the COVID-19 pandemic had an impact on the mental health of the global population. Lockdown periods were associated with a deterioration in population-level mental health, reflecting the extent of mood disturbances triggered by the public health crisis [53]. Studies conducted among young adults similarly demonstrated a high prevalence of psychological symptoms and showed that structured interventions can enhance psychological adaptability and reduce symptom severity [54]. Moreover, during later stages of the pandemic, persistently elevated levels of depression, anxiety, and stress were observed, highlighting the enduring character of its psychological consequences [55]. In this context, our findings indicating mood improvement following psychobiotic supplementation in individuals recovering from COVID-19 provide an important contribution to the development of supportive strategies aimed at improving post-infectious mental health.

In clinical studies assessing probiotic supplementation, both individuals with diagnosed depression or subthreshold depressive symptoms and healthy volunteers were included, in whom effects on mood, stress, and cognitive functioning were evaluated.

Several of these trials used probiotic strains different from those applied in our study, such as *Bifidobacterium bifidum* BGN4 and *Bifidobacterium longum* BORI [42], *Lactobacillus plantarum* JYLP-326 [46], or *Lactobacillus gasseri* CP2305 [51]. However, in clinical trials conducted in patients with depression, the same two probiotic strains (*Lactobacillus helveticus* and *Bifidobacterium longum*) were examined. The use of psychobiotics in the treatment of depression has been examined both as an adjunct to standard antidepressant therapy and as a standalone intervention.

Kazemi et al. evaluated the effect of administering a probiotic and a prebiotic on the psychological symptoms of patients with depression who were receiving antidepressant medications [51]. The study included 110 patients with mild or moderate depression who had been on antidepressant medications—sertraline, fluoxetine, citalopram or amitriptyline—for at least three months prior to the study. Patients were randomly assigned to the following groups: the first group received a probiotic (*Lactobacillus helveticus* Rosell^®^-52 and *Bifidobacterium longum* Rosell^®^-175), the second received a prebiotic (galactooligosaccharide), and the third received a placebo. The supplementation period lasted eight weeks. In individuals receiving the probiotic, a significant reduction in the number of points on the BDI scale was observed compared to both the prebiotic and placebo groups, along with a significant decrease in the ratio of kynurenine (one of the tryptophan metabolites, whose synthesis increases in people with depression) to tryptophan and an increase in the ratio of tryptophan to isoleucine compared to the placebo group. As the authors of the study explained, the reduction in symptom severity reported by patients on the BDI scale may be attributable to increased serotonin production resulting from changes in the tryptophan pathway. Another explanation of the mechanism of action of psychobiotics came from further analysis of the biological material obtained during the study [52]. This analysis showed a significant increase in BDNF concentration among patients receiving *Lactobacillus helveticus* Rosell^®^-52 and *Bifidobacterium longum* Rosell^®^-175 supplementation. It is worth noting that BDNF is a neurotrophin that supports neuronal survival, enhances brain neuroplasticity, and regulates neurogenesis by modulating cell growth, proliferation, migration, differentiation, and cell death.

Ullah et al. studied 80 individuals diagnosed with subthreshold depression and evaluated the effects of psychobiotics and S-adenosylmethionine [50]. The experiment was conducted in a crossover design: after randomization, patients in the first group initially received a combination of S-adenosylmethionine (SAMe, 200 mg/day) and probiotics (*Lactobacillus helveticus* Rosell^®^-52 and *Bifidobacterium longum* Rosell^®^-175; 3 × 10^9^ CFU/day), followed by a placebo, whereas those in the second group received the interventions in the reverse order—first placebo, then the supplement. After six weeks of supplementation with S-adenosylmethionine and psychobiotics, a significant decrease in scores on the Hamilton Depression Rating Scale and the Patient Health Questionnaire-9 was observed, which directly translated into an alleviation of depressive symptoms in the patients and an improvement in their mood.

There are two studies assessing the effects of supplementation with *Lactobacillus helveticus* Rosell^®^-52 and *Bifidobacterium longum* Rosell^®^-175 on depressive symptoms in patients who were not taking antidepressants [56,57]. After 4 weeks of psychobiotic intake, a reduction in scores on various assessment scales was observed: depressed mood (MADRS—Montgomery–Asberg Depression Rating Scale; QUIDS-SR16—a rapid inventory of depressive symptoms), stress intensity (PSQI—Pittsburgh Sleep Quality Index), anhedonia (SHAPS—Snaith-Hamilton Pleasure Scale), and anxiety (GAD-7 and STAI—State-Trait Anxiety Inventory). Importantly, these results persisted at the 8-week mark. As the authors of the manuscript emphasized, the findings clearly indicate the role of probiotics in alleviating depressive symptoms in patients with moderate depression while concurrently underscoring the safety of their use.

It should, however, be noted that not all studies unequivocally demonstrate the beneficial effects of psychobiotics in the treatment of depression. In a study conducted by Gawlik-Kotelnicka et al., 116 pharmacologically treated patients with at least moderate depression were enrolled, with supplementation administered either a preparation containing *Lactobacillus helveticus* Rosell^®^-52 and *Bifidobacterium longum* Rosell^®^-175 or placebo [58]. After 60 days of supplementation, no significant differences were observed in the psychological tests assessing the severity of depressive symptoms (Montgomery–Asberg Depression Rating Scale and the Depression, Anxiety, and Stress Scale). A higher rate of minimal clinically important differences (measured by Cohen’s d score) was, however, noted among the patients receiving probiotic supplementation, leading the study authors to advocate for the inclusion of psychobiotic therapy as an adjunct treatment in cases of depressive disorders.

The mechanisms responsible for the positive effects of psychobiotics are not yet fully understood. It is suggested that they may involve immunomodulatory activity and regulation of inflammatory pathways, including changes in LPS, IL-1β, IL-10, and TNF-α levels [59]. Psychobiotics may also contribute to the production and metabolism of neurotransmitters, modulate tryptophan metabolism, increase the synthesis of short-chain fatty acids (SCFAs), and enhance gut barrier integrity [60]. These processes may collectively influence brain function. In an in vitro study using the Simulator of the Human Intestinal Microbial Ecosystem (SHIME), the combined addition of *Lactobacillus helveticus* R0052 and *Bifidobacterium longum* R0175 increased gamma-aminobutyric acid (GABA) concentrations and altered the cytokine profile, characterized by an increased IL-6/IL-10 ratio and reduced TNF-α levels [61]. These findings suggest that these strains may enhance GABA availability within the intestinal environment. In an animal model, *Bifidobacterium longum* R0175 reduced depressive-like behavior and attenuated caspase-3 activation in the brain following myocardial infarction in rats, indicating neuroprotective and anti-inflammatory effects [62]. Psychobiotic activity is considered part of the broader gut–brain axis, involving bidirectional communication between the gastrointestinal tract and the central nervous system [21].

Our study showed that supplementation with the psychobiotics *Lactobacillus helveticus* Rosell^®^-52 and *Bifidobacterium longum* Rosell^®^-175 significantly increased butyric acid levels. Furthermore, in the PG, a correlation was observed between changes in SCFA concentrations and a reduction in the severity of anxiety symptoms measured by the HADS-A test. The lack of confirmation of this result with other tests may be due to the varying power and sensitivity of the psychological tools used.

Many studies have demonstrated significant interactions between depressive or anxiety episodes and gut microbiota metabolism [63]. SCFAs are natural ligands for free fatty acid receptors 2 and 3 (FFAR2 and FFAR3), which are widely expressed in enteroendocrine, immune, and neural cells [63,64]. The majority of circulating SCFAs originate from gut microbial fermentation and are absorbed in the large intestine. Some studies suggest that their concentrations may increase following probiotic supplementation. SCFAs regulate the transport of nutrients and molecules involved in maintaining blood–brain barrier integrity, thereby directly influencing brain development and central nervous system homeostasis [65]. Furthermore, SCFAs modulate the hypothalamic–pituitary–adrenal axis and tryptophan metabolism [66,67].

Among SCFAs, butyrate is the most potent and the most widely studied in neuropsychiatric research due to its role as a histone deacetylase (HDAC) inhibitor [68]. Schiweck et al.’s study demonstrated lower baseline plasma levels of butyrate and propionate in patients with major depressive disorder compared with healthy controls. Additionally, higher baseline butyrate levels were associated with a greater likelihood of remission at follow-up [69].

Also, in patients with ulcerative colitis, 12 weeks of sodium butyrate supplementation significantly reduced symptoms of depression and anxiety. Butyrate is a potent histone deacetylase (HDAC) inhibitor that promotes the expression of genes that support neuronal plasticity, including BDNF [70].

These findings indicate an association between changes in fecal SCFA concentrations and improvements in anxiety symptoms. However, because fecal SCFA concentrations do not necessarily reflect systemic bioavailability or circulating SCFA levels, these correlations should not be interpreted as evidence of a causal relationship. Further studies are warranted to clarify the underlying mechanisms.

Our study has certain limitations. First, no analysis of the gut microbiota composition was performed. Therefore, we cannot determine whether the observed changes in fecal SCFA concentrations resulted from alterations in the gut microbiota composition, nor can we infer the underlying mechanisms linking psychobiotic supplementation, SCFA production, and improvements in psychological symptoms. Second, stress intensity was assessed solely through self-report measures, reflecting patients’ internal perceptions, and was not verified by laboratory markers (e.g., cortisol levels). Third, the study included only people with symptoms of anxiety–depressive disorders, not patients with severe depression, which allows us to only conclude that the administration of psychobiotics is helpful in alleviating the symptoms of anxiety–depressive disorders, but not in the treatment of severe forms of this disease.

The study was conducted with a relatively small sample size and did not include a healthy control group receiving psychobiotic supplementation, due to cost-related constraints. Additionally, the study population consisted primarily of overweight individuals, so the generalizability of the results to normal-weight populations may be limited. Moreover, endocrine-related factors in women, such as menopausal status and menstrual cycle phase, were not controlled, although both are known to influence anxiety and depressive symptom expression.

The strengths of this study include its randomized, double-blind, placebo-controlled design, which minimizes bias and enhances the reliability of the findings; the use of validated psychological assessment tools; a clear intervention protocol employing standardized psychobiotic strains; a high completion rate; and clinically meaningful outcomes encompassing both symptom severity and the prevalence of anxiety and depressive symptoms.

## 5. Conclusions

This study provides novel evidence that psychobiotic supplementation may benefit individuals recovering from COVID-19 who continue to experience anxiety and depressive symptoms. Overall, 6 weeks of supplementation with a mixture of psychobiotics—*Lactobacillus helveticus* Rosell^®^-52 and *Bifidobacterium longum* Rosell^®^-175—in patients who had gone through COVID-19 and have exhibited anxiety and/or depressive symptoms leads to improved mood and a reduction in the intensity of psychological symptoms. The observed changes were associated with changes in short-chain fatty acid concentrations in feces. Future research should examine long-term effects and involve larger, more diverse cohorts to identify individuals most likely to benefit from psychobiotic interventions.

## Figures and Tables

**Table 1:** Anthropometric and demographic data of patients at baseline.

Parameter	Psychobiotic Group (n = 32)		Control Group (n = 30)		*p* *
	Median (Q1–Q3)	Mean ± SD (95% CI)	Median (Q1–Q3)	Mean ± SD (95% CI)	
Age [years]	56 (46–60)	53 ± 11 (49 to 57)	56 (45–59)	49 ± 15 (43 to 55)	0.8056
Body weight [kg]	81.5 (68.3–94.0)	82.6 ± 18.3 (75.9 to 89.3)	77.0 (65.0–90.5)	81.0 ± 23.9 (72.1 to 89.9)	0.4840
Body height [cm]	167 (162–172)	168 ± 11 (164 to 172)	167 (164–173)	168 ± 8 (165 to 171)	0.5934
BMI [kg/m^2^]	29.9 (25.7–32.8)	29.4 ± 4.9 (27.4 to 31.0)	28.7 (23.1–31.1)	28.4 ± 7.1 (25.7 to 31.2)	0.2812
BMI ≥ 25.0 kg/m^2^ [%]	78.1		66.6		0.3201
BMI ≥ 30.0 kg/m^2^ [%]	43.7		33.3		0.7082
Sex: female [%]	71.8		70.0		0.8721
Place of residence					
Village (from the city agglomeration)	15.6		10.0		
a city with fewer than 500 thousand residents	68.8		70.0		0.9077
a city with more than 500 thousand residents	15.6		20.0		
Self-reported financial status					
Low	0.0		0.0		
Middle	18.8		26.7		0.9066
High	81.2		73.3		

* *p*-value for the U Mann–Whitney test or the χ^2^ test. Q—quartile; SD—standard deviation; CI—confidence interval; BMI—body mass index.

**Table 2:** Levels of depression, anxiety, and the global severity index of psychopathological symptoms at baseline and after 6 weeks of supplementation.

	Psychobiotic Group (n = 28)						Control Group (n = 28)					
	Baseline (BL)		Follow-Up (FU)		Δ FU − BL		Baseline (BL)		Follow-Up (FU)		Δ FU − BL	
	Median (Q1–Q3)	Mean ± SD (95% CI)	Median (Q1–Q3)	Mean ± SD (95% CI)	Median (Q1–Q3)	Mean ± SD (95% CI)	Median (Q1–Q3)	Mean ± SD (95% CI)	Median (Q1–Q3)	Mean ± SD (95% CI)	Median (Q1–Q3)	Mean ± SD (95% CI)
**HADS—Depression**	10.0 (8.0–12.0)	9.7 ± 2.9 (8.5–10.8)	5.5 * (1.0–7.2)	4.4 ± 3.2 (3.2–5.6)	**−5.0 ^⁑^** (−6.2–−3.7)	−5.2 ± 2.6 (−6.3–−4.2)	10.0 (8.0–12.2)	10.1 ± 4.1 (8.5–11.7)	8.5 * (6.7–10.2)	8.8 ± 3.7 (7.4–10.3)	**−1.0 ^⁑^** (−2.0–0.0)	−1.2 ± 1.4 (−1.8–0.7)
**HADS—Anxiety**	12.0 (11.5–14.0)	12.0 ± 2.5 (11.0–12.9)	6.0 * (4.0–10.0)	6.6 ± 3.3 (5.3–7.8)	**−5.0 ^⁑^** (−7.0–−3.7)	−5.4 ± 2.5 (−6.4–−4.4)	12.0 (12.0–14.2)	13.2 ± 2.7 (12.2–14.3)	11.0 * (10.0–12.5)	11.7 ± 2.9 (10.5–12.8)	**−2.0 ^⁑^** (−2.0–−1.0)	−1.6 ± 1.3 (−2.1–−1.0)
**GAD-7**	11.5 (10.0–13.0)	11.2 ± 4.0 (9.7–12.8)	4.0 * (2.7–6.2)	4.3 ± 3.1 (3.1–5.5)	**−7.0 ^⁑^** (−9.0–−5.7)	−7.0 ± 3.0 (−8.1–−5.8)	10.0 (6.7–13.0)	10.1 ± 5.4 (7.9–12.2)	8.5 * (6.7–11.2)	9.3 ± 4.3 (7.6–11.0)	**−1.0 ^⁑^** (−2.25–1.0)	−0.8 ± 2.7 (−1.8–0.3)
**BDI**	15.5 (11.0–21.2)	16.4 ± 8.4 (13.1–19.6)	**8.0 †** (2.7–12.2)	8.0 ± 6.2 (5.6–10.4)	**−7.0 ^⁑^** (−11.2–−4.0)	−8.3 ± 4.8 (−10.2–−6.4)	16.0 (11.0–23.2)	18.7 ± 10.5 (14.6–22.7)	**14.5 †** (11.5–19.2)	16.1 ± 8.7 (12.7–19.5)	**−2.0 ^⁑^** (−4.0–0.0)	−2.6 ± 4.0 (−4.1–−1.0)
**SCL-90** **Global severity index**	11.0 (6.8–12.6)	10.5 ± 4.6 (8.7–12.3)	**6.3 ^※^** (3.5–8.7)	6.5 ± 3.5 (5.1–7.8)	**−4.1 ^⁂^** (−5.7–−2.0)	−4.0 ± 2.4 (−4.9–−3.1)	9.7 (8.5–13.7)	11.7 ± 5.1 (9.7–13.7)	**8.0 ^※^** (6.6–12.0)	10.2 ± 5.5 (8.0–12.3)	**−1.4 ^⁂^** (−2.5–−0.3)	−1.5 ± 1.9 (−2.2–−0.7)

Q—quartile, SD—standard deviation, CI—confidence interval; HADS—Hospital Anxiety and Depression Scale; GAD-7—Generalized Anxiety Disorder-7; BDI—Beck Depression Inventory; SCL-90—Polish version of the HSCL 90, Hopkins Symptom Checklist. Follow-up vs. Follow-up *p* value: * 0.0001, † 0.0003, **^※^** 0.01; Δ FU − BL vs. Δ FU − BL ^⁑^ < 0.0001, ^⁂^ < 0.0002.

**Table 3:** Severity of psychopathological symptoms measured with the Polish version of the Hopkins Symptom Checklist at baseline and after 6 weeks of supplementation.

	Psychobiotic Group (n = 28)						Control Group (n = 28)					
	Baseline (BL)		Follow-Up (FU)		Δ FU − BL		Baseline (BL)		Follow-Up (FU)		Δ FU − BL	
	Median (Q1–Q3)	Mean ± SD (95% CI)	Median (Q1–Q3)	Mean ± SD (95% CI)	Median (Q1–Q3)	Mean ± SD (95% CI)	Median (Q1–Q3)	Mean ± SD (95% CI)	Median (Q1–Q3)	Mean ± SD (95% CI)	Median (Q1–Q3)	Mean ± SD (95% CI)
Somatization	1.7 (1.0–2.4)	1.7 ± 0.8 (1.4–2.1)	1.2 (0.7–1.5)	1.2 ± 0.7 (0.9–1.5)	−0.4 (−1.1–0.0)	−0.5 ± 0.6 (−0.8–−0.3)	1.8 (1.2–2.1)	1.8 ± 0.8 (1.5–2.1)	1.3 (1.00–1.9)	1.5 ± 0.8 (1.2–1.9)	0.0 (−0.5–0.0)	−0.2 ± 0.4 (−0.4–−0.1)
Obsessive- -compulsive	1.7 (1.3–2.1)	1.7 ± 0.7 (1.4–2.0)	1.2 (0.7–1.6)	1.2 ± 0.6 (1.0–1.4)	−0.3 (−0.8–−0.1)	−0.5 ± 0.5 (−0.7–−0.3)	1.6 (1.3–2.1)	1.7 ± 0.8 (1.4–2.0)	1.3 (1.0–1.8)	1.5 ± 0.8 (1.2–1.8)	−0.2 (−0.5–0.0)	−0.2 ± 0.4 (−0.4–−0.1)
Interpersonal sensitivity	0.8 (0.4–1.1)	0.9 ± 0.8 (0.6–1.2)	0.6 * (0.2–0.7)	0.6 ± 0.5 (0.4–0.8)	−0.2 (−0.5–0.0)	−0.4 ± 0.4 (−0.5–−0.2)	1.1 (0.8–2.0)	1.4 ± 0.9 (1.0–1.8)	0.9 * (0.7–1.7)	1.2 ± 0.8 (0.9–1.5)	−0.1 (−0.3–0.0)	−0.2 ± 0.4 (−0.4–0.0)
Depression	1.3 (1.0–1.7)	1.4 ± 0.7 (1.2–1.7)	**0.5 †** (0.3–0.9)	0.7 ± 0.5 (0.5–0.9)	**−0.6 ^⁑^** (−1.0–−0.5)	−0.7 ± 0.4 (−0.9–−0.6)	1.5 (1.1–2.1)	1.7 ± 0.8 (1.3–2.0)	**1.2 †** (0.9–1.8)	1.4 ± 0.8 (1.2–1.8)	**−0.1 ^⁑^** (0.0–−0.1)	−0.2 ± 0.3 (−0.3–−0.1)
Anxiety	1.5 (1.0–2.1)	1.6 ± 0.6 (1.3–1.8)	**0.6 †** (0.3–1.0)	0.7 ± 0.5 (0.5–0.9)	**−0.8 ^⁑^** (−1.1–−0.5)	−0.9 ± 0.4 (−1.0–−0.7)	1.4 (1.1–2.0)	1.6 ± 0.6 (1.4–1.9)	**1.3 †** (1.0–1.7)	1.4 ± 0.6 (1.2–1.7)	**−0.1 ^⁑^** (−0.2–−0.1)	−0.2 ± 0.2 (−0.3–−0.1)
Anger–hostility	1.1 (0.4–1.3)	1.1 ± 0.7 (0.8–1.3)	0.7 (0.3–0.8)	0.7 ± 0.4 (0.5–0.9)	−0.3 (−0.5–−0.1)	−0.4 ± 0.4 (−0.5–−0.3)	1.0 (0.6–1.3)	1.0 ± 0.6 (0.8–1.3)	0.7 (0.3–1.1)	0.9 ± 0.7 (0.6–1.2)	−0.2 (−0.3–0.0)	−0.1 ± 0.4 (−0.3–0.0)
Phobic anxiety	0.4 (0.3–0.9)	0.6 ± 0.6 (0.4–0.9)	0.4 (0.1–0.5)	0.4 ± 0.3 (0.3–0.5)	−0.1 (−0.3–0.0)	−0.2 ± 0.4 (−0.4–−0.1)	0.5 (0.2–1.3)	0.7 ± 0.7 (0.4–1.0)	0.3 (0.2–1.0)	0.6 ± 0.6 (0.3–0.9)	0.0 (−0.2–0.0)	−0.1 ± 0.2 (−0.2–0.0)
Paranoid ideation	1.0 (0.5–1.3)	0.8 ± 0.5 (0.6–1.0)	0.5 * (0.3–0.8)	0.6 ± 0.5 (0.4–0.8)	−0.1 (−0.5–0.0)	−0.2 ± 0.4 (−0.4–−0.1)	1.0 (0.7–1.3)	1.0 ± 0.5 (0.8–1.2)	0.8 * (0.7–1.1)	0.9 ± 0.5 (0.7–1.1)	−0.1 (−0.2–0.0)	−0.1 ± 0.4 (−0.2–0.1)
Psychoticism	0.5 (0.3–0.8)	0.5 ± 0.3 (0.4–0.6)	0.3 (0.2–0.5)	0.4 ± 0.3 (0.2–0.5)	0.0 (−0.2–0.0)	−0.1 ± 0.2 (−0.2–0.0)	0.5 (0.4–0.9)	0.6 ± 0.3 (0.5–0.7)	0.5 (0.3–0.7)	0.5 ± 0.3 (0.4–0.6)	0.0 (−0.2–0.0)	−0.1 ± 0.1 (−0.1–0.0)

Q—quartile; SD—standard deviation; CI—confidence interval. * Significant difference between groups at follow-up (*p* = 0.001). **†** Significant difference between groups at follow-up (*p* < 0.0001). **⁑** Significant difference in change from baseline (Δ follow-up − baseline) between groups (*p* < 0.0001).

**Table 4:** Number of patients in the treatment and control groups with a diagnosis of depression or anxiety at baseline and after 6 weeks of supplementation.

	Psychobiotic Group (n = 28)		Control Group (n = 28)	
	Baseline	Follow-Up	Baseline	Follow-Up
HADS—Depression	12	0	13	7
HADS—Anxiety	21 *	4 *	27 *	15 *
GAD-7	22 *	2 *	15 *	10 *
BDI	18 *	8 *	20 *	21 *
SCL –Somatization	13	4	8	7
SCL—Obsessive–compulsive	8	2	11	6
SCL—Interpersonal sensitivity	3	0	9	7
SCL—Depression	7	1	8	5
SCL—Anxiety	11	0	8	5
SCL—Anger–hostility	3	1	3	3
SCL—Phobic anxiety	2	0	2	2
SCL—Paranoid ideation	0	0	0	0
SCL—Psychoticism	0	0	0	0

* statistically significant differences. HADS—Hospital Anxiety and Depression Scale; GAD-7—Generalized Anxiety Disorder-7; BDI—Beck Depression Inventory; SCL—Polonized version of the Hopkins Symptom Checklist.

**Table 5:** Fecal SCFA concentration [μmol/g] at baseline and after 6 weeks of supplementation.

	Psychobiotic Group (n = 28)						Control Group (n = 28)					
	Baseline (BL)		Follow-Up (FU)		Δ FU − BL		Baseline (BL)		Follow-Up (FU)		Δ FU − BL	
	Median (Q1–Q3)	Mean ± SD (95% CI)	Median (Q1–Q3)	Mean ± SD (95% CI)	Median (Q1–Q3)	Mean ± SD (95% CI)	Median (Q1–Q3)	Mean ± SD (95% CI)	Median (Q1–Q3)	Mean ± SD (95% CI)	Median (Q1–Q3)	Mean ± SD (95% CI)
Acetic acid [μmol/g]	26.2 (16.6–40.3)	28.9 ± 16.0 (22.7–35.1)	32.5 (17.3–40.4)	34.7 ± 21.5 (26.4–31.1)	3.3 (−6.9–19.1)	5.8 ± 20.8 (−2.2–13.9)	26.9 (22.8–33.0)	30.8 ± 14.9 (25.0–36.6)	23.0 (18.2–31.9)	27.4 ± 12.3 (22.6–32.2)	1.3 (−8.4–7.5)	−3.4 ± 17.9 (−10.4–3.5)
Propionic acid [μmol/g]	10.9 (5.1–14.7)	10.8 ± 6.7 (8.2–13.4)	10.0 (5.7–18.5)	13.1 ± 9.7 (9.3–16.8)	0.4 (−2.8–4.1)	2.3 ± 9.1 (−1.2–5.8)	9.1 (5.3–15.7)	11.5 ± 9.7 (7.8–5.3)	8.4 (4.6–11.8)	9.3 ± 6.1 (6.9–11.7)	−0.3 (−5.9–3.6)	−2.2 ± 9.1 (−5.7–1.3)
Butyric acid [μmol/g]	1.8 * (0.7–4.4)	3.6 ± 4.4 (1.9–5.4)	4.2 *^※^ (2.1–6.4)	6.2 ± 6.4 (3.6–8.7)	**1.5 ^⁑^** (−0.1–4.0)	2.5 ± 5.8 (0.3–4.8)	3.5 (0.9–5.5)	4.6 ± 4.8 (2.7–6.4)	**2.0 ^※^** (0.8–5.1)	3.0 ± 3.0 (1.8–4.2)	**−0.1 ^⁑^** (−4.6–1.0)	−1.6 ± 4.6 (−3.3–0.2)

Q—quartile; SD—standard deviation; CI—confidence interval, Baseline vs. Follow-up * < 0.05; Follow-up vs. Follow-up **^※^ <** 0.05; Δ FU − BL vs. Δ FU − BL ^⁑^ < 0.01.

## Data Availability

The data presented in this study are available from the corresponding author upon request. The data are not publicly available due to ethical restrictions, in particular those related to the protection of personal data and the privacy of the study participants.

## References

[1] Manchia M., Gathier A.W., Yapici-Eser H., Schmidt M.V., De Quervain D., Van Amelsvoort T., Bisson J.I., Cryan J.F., Howes O.D., Pinto L. (2022). The Impact of the Prolonged COVID-19 Pandemic on Stress Resilience and Mental Health: A Critical Review across Waves. Eur. Neuropsychopharmacol..

[2] Pasha A., Inusah A.-H.S., Nayem J., Li X., Qiao S. (2026). Psychological Resilience in the COVID-19 Response: A Systematic Review for Future Public Health Preparedness. Curr. Psychol..

[3] Miraj S.S., Ali S.A. (2023). Perspectives for Better Health: Prepare for the Exiting Severe Phase of the COVID-19 Pandemic. J. Med. Sci..

[4] Ayyala R.S. (2022). Coronavirus Disease 2019 (COVID-19) and Physicians with a Disability: A Compounding Stressor. Pediatr. Radiol..

[5] Hosseini N., Nadjafi S., Ashtary B. (2021). Overview of COVID-19 and Neurological Complications. Rev. Neurosci..

[6] Castro-de-Araujo L.F.S., Rodrigues E.D.S., Machado D.B., Henriques C.M.P., Verotti M.P., Gonçalves A.Q., Duarte-Salles T., Kanaan R.A., Barreto M.L., Lewis G. (2022). Multimorbidity Worsened Anxiety and Depression Symptoms during the COVID-19 Pandemic in Brazil. J. Affect. Disord..

[7] Haidar M., Shakkour Z., Reslan M., Al-Haj N., Chamoun P., Habashy K., Kaafarani H., Shahjouei S., Farran S., Shaito A. (2022). SARS-CoV-2 Involvement in Central Nervous System Tissue Damage. Neural Regen. Res..

[8] Mateescu D.-M., Ilie A.-C., Cotet I., Guse C., Muresan C.-O., Pah A.-M., Badalica-Petrescu M., Iurciuc S., Craciun M.-L., Avram A. (2025). Gut Microbiome Dysbiosis in COVID-19: A Systematic Review and Meta-Analysis of Diversity Indices, Taxa Alterations, and Mortality Risk. Microorganisms.

[9] Natarajan A., Zlitni S., Brooks E.F., Vance S.E., Dahlen A., Hedlin H., Park R.M., Han A., Schmidtke D.T., Verma R. (2022). Gastrointestinal Symptoms and Fecal Shedding of SARS-CoV-2 RNA Suggest Prolonged Gastrointestinal Infection. Med.

[10] Al-Momani H., Aolymat I., Almasri M., Mahmoud S.A., Mashal S. (2023). Prevalence of Gastrointestinal Symptoms Among COVID-19 Patients and the Association With Disease Clinical Outcomes. Future Sci. OA.

[11] Arruda I.D.S.A., Cavalcante C.D.S., Rubens R.S., Castro L.N.P.D.F., Nóbrega Y.K.D.M., Dalmolin T.V. (2025). Changes in the Gut Microbiota of Patients After SARS-CoV-2 Infection: What Do We Know?. Microorganisms.

[12] Doroszkiewicz J., Groblewska M., Mroczko B. (2021). The Role of Gut Microbiota and Gut–Brain Interplay in Selected Diseases of the Central Nervous System. Int. J. Mol. Sci..

[13] Sonali S., Ray B., Ahmed Tousif H., Rathipriya A.G., Sunanda T., Mahalakshmi A.M., Rungratanawanich W., Essa M.M., Qoronfleh M.W., Chidambaram S.B. (2022). Mechanistic Insights into the Link between Gut Dysbiosis and Major Depression: An Extensive Review. Cells.

[14] Nozari N. (2021). COVID-19 Outbreak and Its Burden on a New Wave of Functional Gastrointestinal Disorders. Middle East J. Dig. Dis..

[15] Dinan T.G., Stanton C., Cryan J.F. (2013). Psychobiotics: A Novel Class of Psychotropic. Biol. Psychiatry.

[16] Dinan T.G., Cryan J.F. (2017). Brain-Gut-Microbiota Axis and Mental Health. Psychosom. Med..

[17] Monteagudo-Mera A., Rastall R.A., Gibson G.R., Charalampopoulos D., Chatzifragkou A. (2019). Adhesion Mechanisms Mediated by Probiotics and Prebiotics and Their Potential Impact on Human Health. Appl. Microbiol. Biotechnol..

[18] Sori N., Khan M. (2024). Gamma Amino Butyric Acid (GABA) and Ferulic Acid Esterase (FAE) Producing Psychobiotic Bacteria Isolated from Cereal-Based Fermented Food. Curr. Microbiol..

[19] Colletti A., Pellizzato M., Cicero A.F. (2023). The Possible Role of Probiotic Supplementation in Inflammation: A Narrative Review. Microorganisms.

[20] Dinan T.G., Cryan J.F. (2017). The Microbiome-Gut-Brain Axis in Health and Disease. Gastroenterol. Clin. N. Am..

[21] Sarkar A., Lehto S.M., Harty S., Dinan T.G., Cryan J.F., Burnet P.W.J. (2016). Psychobiotics and the Manipulation of Bacteria–Gut–Brain Signals. Trends Neurosci..

[22] Bambury A., Sandhu K., Cryan J.F., Dinan T.G. (2018). Finding the Needle in the Haystack: Systematic Identification of Psychobiotics. Br. J. Pharmacol..

[23] Carpenter A., Carnovale M., Davies J., De Cicco M., Henoud S., Laulund S., Naderi N., Ng W., Sewalt V. (2026). Comprehensive Review of Safe Microbial Ingredients for Use in Food. J. Food Prot..

[24] Misra S., Mohanty D. (2019). Psychobiotics: A New Approach for Treating Mental Illness?. Crit. Rev. Food Sci. Nutr..

[25] Nowak D.J. (2024). Study Designs in Medical Research and Their Key Characteristics. J. Med. Sci..

[26] Ricci A., Allende A., Bolton D., Chemaly M., Davies R., Girones R., Koutsoumanis K., Lindqvist R., Nørrung B., EFSA Panel on Biological Hazards (BIOHAZ) (2017). Update of the List of QPS-recommended Biological Agents Intentionally Added to Food or Feed as Notified to EFSA 6: Suitability of Taxonomic Units Notified to EFSA until March 2017. EFS2.

[27] Bourdichon F., Casaregola S., Farrokh C., Frisvad J.C., Gerds M.L., Hammes W.P., Harnett J., Huys G., Laulund S., Ouwehand A. (2012). Food Fermentations: Microorganisms with Technological Beneficial Use. Int. J. Food Microbiol..

[28] Zigmond A.S., Snaith R.P. (1983). The Hospital Anxiety and Depression Scale. Acta Psychiatr. Scand..

[29] Baldwin D., Woods R., Lawson R., Taylor D. (2011). Efficacy of Drug Treatments for Generalised Anxiety Disorder: Systematic Review and Meta-Analysis. BMJ.

[30] Cuijpers P., Sijbrandij M., Koole S., Huibers M., Berking M., Andersson G. (2014). Psychological Treatment of Generalized Anxiety Disorder: A Meta-Analysis. Clin. Psychol. Rev..

[31] Beck A.T. (1961). An Inventory for Measuring Depression. Arch. Gen. Psychiatry.

[32] Richter P., Werner J., Heerlein A., Kraus A., Sauer H. (1998). On the Validity of the Beck Depression Inventory. Psychopathology.

[33] Kuncewicz D., Dragan M., Hardt J. (2014). Validation of the Polish version of the symptom checklist-27-plus questionnaire. Psychiatr. Pol..

[34] Derogatis L.R., Lipman R.S., Rickels K., Uhlenhuth E.H., Covi L., Pichot P., Olivier-Martin R. (1974). The Hopkins Symptom Checklist (HSCL). Modern Trends in Pharmacopsychiatry.

[35] Pieczyńska-Zając J.M., Malinowska A.M., Pruszyńska-Oszmałek E., Kołodziejski P.A., Drzymała-Czyż S., Bajerska J. (2024). Effect of a High-Fat High-Fructose Diet on the Composition of the Intestinal Microbiota and Its Association with Metabolic and Anthropometric Parameters in a Letrozole-Induced Mouse Model of Polycystic Ovary Syndrome. Nutrition.

[36] Hopewell S., Chan A.-W., Collins G.S., Hróbjartsson A., Moher D., Schulz K.F., Tunn R., Aggarwal R., Berkwits M., Berlin J.A. (2025). CONSORT 2025 Statement: Updated Guideline for Reporting Randomised Trials. BMJ.

[37] Kupcova I., Danisovic L., Klein M., Harsanyi S. (2023). Effects of the COVID-19 Pandemic on Mental Health, Anxiety, and Depression. BMC Psychol..

[38] Drzymała S., Blask-Osipa A., Szczepańska-Alvarez A., Markowska H., Dobrzyńska M., Drzymała-Czyż S., Walkowiak J. (2026). Anxiety-Depressive Disorders in 200 Patients with Post-COVID-19 Syndrome: Prevalence and Predictors from a Cross-Sectional Study. Medicina.

[39] Shetty P.A., Ayari L., Madry J., Betts C., Robinson D.M., Kirmani B.F. (2023). The Relationship Between COVID-19 and the Development of Depression: Implications on Mental Health. J. Exp. Neurosci..

[40] Santomauro D.F., Mantilla Herrera A.M., Shadid J., Zheng P., Ashbaugh C., Pigott D.M., Abbafati C., Adolph C., Amlag J.O., Aravkin A.Y. (2021). Global Prevalence and Burden of Depressive and Anxiety Disorders in 204 Countries and Territories in 2020 Due to the COVID-19 Pandemic. Lancet.

[41] Ivanov I., Schwartz J.M. (2021). Why Psychotropic Drugs Don’t Cure Mental Illness—But Should They?. Front. Psychiatry.

[42] Kim C.-S., Cha J., Sim M., Jung S., Chun W.Y., Baik H.W., Shin D.-M. (2021). Probiotic Supplementation Improves Cognitive Function and Mood with Changes in Gut Microbiota in Community-Dwelling Older Adults: A Randomized, Double-Blind, Placebo-Controlled, Multicenter Trial. J. Gerontol. Ser. A.

[43] Ho Y.-T., Tsai Y.-C., Kuo T.B.J., Yang C.C.H. (2021). Effects of Lactobacillus Plantarum PS128 on Depressive Symptoms and Sleep Quality in Self-Reported Insomniacs: A Randomized, Double-Blind, Placebo-Controlled Pilot Trial. Nutrients.

[44] Boehme M., Rémond-Derbez N., Lerond C., Lavalle L., Keddani S., Steinmann M., Rytz A., Dalile B., Verbeke K., Van Oudenhove L. (2023). Bifidobacterium Longum Subsp. Longum Reduces Perceived Psychological Stress in Healthy Adults: An Exploratory Clinical Trial. Nutrients.

[45] Messaoudi M., Lalonde R., Violle N., Javelot H., Desor D., Nejdi A., Bisson J.-F., Rougeot C., Pichelin M., Cazaubiel M. (2011). Assessment of Psychotropic-like Properties of a Probiotic Formulation (*Lactobacillus Helveticus* R0052 and *Bifidobacterium Longum* R0175) in Rats and Human Subjects. Br. J. Nutr..

[46] Messaoudi M., Violle N., Bisson J.-F., Desor D., Javelot H., Rougeot C. (2011). Beneficial Psychological Effects of a Probiotic Formulation (*Lactobacillus Helveticus* R0052 and *Bifidobacterium Longum* R0175) in Healthy Human Volunteers. Gut Microbes.

[47] Diop L., Guillou S., Durand H. (2008). Probiotic Food Supplement Reduces Stress-Induced Gastrointestinal Symptoms in Volunteers: A Double-Blind, Placebo-Controlled, Randomized Trial. Nutr. Res..

[48] Zhu R., Fang Y., Li H., Liu Y., Wei J., Zhang S., Wang L., Fan R., Wang L., Li S. (2023). Psychobiotic Lactobacillus Plantarum JYLP-326 Relieves Anxiety, Depression, and Insomnia Symptoms in Test Anxious College via Modulating the Gut Microbiota and Its Metabolism. Front. Immunol..

[49] Nishida K., Sawada D., Kuwano Y., Tanaka H., Rokutan K. (2019). Health Benefits of Lactobacillus Gasseri CP2305 Tablets in Young Adults Exposed to Chronic Stress: A Randomized, Double-Blind, Placebo-Controlled Study. Nutrients.

[50] Ullah H., Di Minno A., Esposito C., El-Seedi H.R., Khalifa S.A.M., Baldi A., Greco A., Santonastaso S., Cioffi V., Sperandeo R. (2022). Efficacy of a Food Supplement Based on S-Adenosyl Methionine and Probiotic Strains in Subjects with Subthreshold Depression and Mild-to-Moderate Depression: A Monocentric, Randomized, Cross-over, Double-Blind, Placebo-Controlled Clinical Trial. Biomed. Pharmacother..

[51] Kazemi A., Noorbala A.A., Azam K., Eskandari M.H., Djafarian K. (2019). Effect of Probiotic and Prebiotic vs Placebo on Psychological Outcomes in Patients with Major Depressive Disorder: A Randomized Clinical Trial. Clin. Nutr..

[52] Heidarzadeh-Rad N., Gökmen-Özel H., Kazemi A., Almasi N., Djafarian K. (2020). Effects of a Psychobiotic Supplement on Serum Brain-Derived Neurotrophic Factor Levels in Depressive Patients: A Post Hoc Analysis of a Randomized Clinical Trial. J. Neurogastroenterol. Motil..

[53] Wang J., Zhang X., Deng H., Tan Y. (2025). The Effect of the COVID-19 Pandemic Lockdown on Self-Harm: A Meta-Analysis. Alpha Psychiatry.

[54] Onur Ç., Karaaziz M. (2024). Resilience of University Students During the Coronavirus Disease 2019 Pandemic and Results of a Pilot Positive Psychotherapy Intervention Study. Alpha Psychiatry.

[55] Ahmed M.Z., Ahmed O., Hanbin S., Xie P., Jobe M.C., Li W. (2024). Depression, Anxiety, and Stress Among Chinese People During the Omicron Outbreak and Its Impact on Sleep Quality and Alcohol Dependency. Alpha Psychiatry.

[56] Wallace C.J.K., Foster J.A., Soares C.N., Milev R.V. (2020). The Effects of Probiotics on Symptoms of Depression: Protocol for a Double-Blind Randomized Placebo-Controlled Trial. Neuropsychobiology.

[57] Wallace C.J.K., Milev R.V. (2021). The Efficacy, Safety, and Tolerability of Probiotics on Depression: Clinical Results From an Open-Label Pilot Study. Front. Psychiatry.

[58] Gawlik-Kotelnicka O., Margulska A., Płeska K., Skowrońska A., Strzelecki D. (2024). Metabolic Status Influences Probiotic Efficacy for Depression—PRO-DEMET Randomized Clinical Trial Results. Nutrients.

[59] Wiącek J., Skonieczna-Żydecka K., Łoniewski I., Deli C.K., Fatouros I.G., Jamurtas A.Z., Moszczyńska D., Karolkiewicz J. (2025). Lactobacillus Helveticus R0052 and Bifidobacterium Longum R0175 Supplementation: An Exploratory, Randomized, Placebo-Controlled Trial of Endocannabinoid and Inflammatory Responses in Female Dancers. Microorganisms.

[60] Kamal N., Saharan B.S., Duhan J.S., Kumar A., Chaudhary P., Goyal C., Kumar M., Goyat N., Sindhu M., Mudgil P. (2025). Exploring the Promise of Psychobiotics: Bridging Gut Microbiota and Mental Health for a Flourishing Society. Med. Microecol..

[61] De Oliveira F.L., Salgaço M.K., De Oliveira M.T., Mesa V., Sartoratto A., Peregrino A.M., Ramos W.S., Sivieri K. (2023). Exploring the Potential of Lactobacillus Helveticus R0052 and Bifidobacterium Longum R0175 as Promising Psychobiotics Using SHIME. Nutrients.

[62] Trudeau F., Gilbert K., Tremblay A., Tompkins T.A., Godbout R., Rousseau G. (2019). Bifidobacterium Longum R0175 Attenuates Post-Myocardial Infarction Depressive-like Behaviour in Rats. PLoS ONE.

[63] Cheng J., Hu H., Ju Y., Liu J., Wang M., Liu B., Zhang Y. (2024). Gut Microbiota-derived Short-chain Fatty Acids and Depression: Deep Insight into Biological Mechanisms and Potential Applications. Gen. Psychiatry.

[64] Kimura I., Ichimura A., Ohue-Kitano R., Igarashi M. (2020). Free Fatty Acid Receptors in Health and Disease. Physiol. Rev..

[65] Fock E., Parnova R. (2023). Mechanisms of Blood–Brain Barrier Protection by Microbiota-Derived Short-Chain Fatty Acids. Cells.

[66] Agus A., Clément K., Sokol H. (2021). Gut Microbiota-Derived Metabolites as Central Regulators in Metabolic Disorders. Gut.

[67] Tiwari S., Paramanik V. (2025). Role of Probiotics in Depression: Connecting Dots of Gut-Brain-Axis Through Hypothalamic-Pituitary Adrenal Axis and Tryptophan/Kynurenic Pathway Involving Indoleamine-2,3-Dioxygenase. Mol. Neurobiol..

[68] Stilling R.M., Van De Wouw M., Clarke G., Stanton C., Dinan T.G., Cryan J.F. (2016). The Neuropharmacology of Butyrate: The Bread and Butter of the Microbiota-Gut-Brain Axis?. Neurochem. Int..

[69] Schiweck C., Dalile B., Balliet A., Aichholzer M., Reinken H., Erhardt F., Freiling J., Bouzouina A., Uckermark C., Reif A. (2025). Circulating Short Chain Fatty Acids Are Associated with Depression Severity and Predict Remission from Major Depressive Disorder. Brain Behav. Immun.-Health.

[70] Korenblik V., Schilder N.K.M., De Lange I.G.S., Daams J.G., Bockting C.L.H., Brul S., Nieuwdorp M., Lok A., Korosi A. (2026). From Gut to Glee: Is Butyrate a Promising Antidepressant? A Systematic Review and Mechanistic Insights. Brain Behav. Immun..

